# Putting the Puzzle Back Together—A Narrative Case Study of an Athlete Who Survived Child Sexual Abuse in Sport

**DOI:** 10.3389/fpsyg.2022.856957

**Published:** 2022-03-29

**Authors:** Allyson Gillard, Elisabeth St-Pierre, Stephanie Radziszewski, Sylvie Parent

**Affiliations:** ^1^Physical Education, Laval University, Quebec, QC, Canada; ^2^Research Chair on Security and Integrity in Sport (SIMS), Laval University, Quebec, QC, Canada; ^3^Interdisciplinary Research Centre on Intimate Relationship Problems and Sexual Abuse (CRIPCAS), Montreal, QC, Canada; ^4^Équipe Violence Sexuelle et Santé (ÉVISSA), Faculty of Social Science, Université du Québec à Montréal, Montréal, QC, Canada; ^5^Faculty of Psychology, Université du Québec à Montréal, Montréal, QC, Canada

**Keywords:** sport, child sexual abuse, sexual abuse, athlete, disclosure, healing, understanding

## Abstract

Denunciations of child sexual abuse (CSA) in the sport context have been increasing in the last decades. Studies estimate that between 14 and 29% of athletes have been victim of at least one form of sexual violence in sport before the age of 18. However, studies suggest that many do not disclose their experience of CSA during childhood. This finding is alarming since studies have shown that the healing process usually starts with disclosure. Moreover, little is known about the healing process of CSA experienced in the sport context. The aim of the study is to present a single case study of a CSA in sport to better understand the global experience over time from the perspective of the athlete. A narrative inquiry approach was adopted. Three non-structured interviews were conducted with the participant. Three pathways in the survivor journey have been identified through inductive thematic analysis: (a) pathway to understanding, (b) pathway to disclosure, and (c) pathway to healing. These pathways represent distinct processes but are intertwined as they are dynamic and iterative. Indeed, the survivor explained how she had been, and is still, going back and forth between them. Results are consistent with those found in the literature on CSA in the general population. It suggests that theoretical models of CSA in the general population could be applied to CSA in sport. Practical implications include a need for education and clearer boundaries in the coach-athlete relationship. Sport stakeholders also need to be better equipped to recognize the signs of sexual violence in sport. Our results indicate that qualitative research could be a potential avenue to help victims heal from CSA. It gives them the chance to talk about and make sense of their abuse in a safe space. Finally, our results demonstrate the importance of reviewing the current justice system for victims. It should be based on a trauma-informed approach that places the victim at the center of the judicial process.

## Introduction

The last few decades have been marked by a growing denunciation of child sexual abuse (CSA) in the sport context. Studies show that 14 to 29% of athletes have been a victim of at least one form of sexual violence before the age of 18 (Alexander et al., 2011; Vertommen et al., 2016; Parent and Vaillancourt-Morel, 2020). Child sexual abuse is defined as “the involvement of a child in sexual activity that he or she does not fully comprehend, is unable to give informed consent to, for which the child is not developmentally prepared and cannot give consent, or that violates the laws or social taboos of society” (World Health Organization, 1999; Woodward and Joseph, 2003, p. 15–16). Child sexual abuse is a threat to the child’s healthy development (Halvorsen et al., 2020) and has many consequences including depression, anxiety, post-traumatic symptoms, eating disorders, and increased risk of suicide (Leahy et al., 2008; Vertommen et al., 2016; Mountjoy, 2019; Timpka et al., 2019; Parent and Vaillancourt-Morel, 2020). While similar consequences have been reported in the general population, there are also sport-specific consequences such as a decrease in performance and giving up sport (Fasting et al., 2002; Bisgaard and Støckel, 2019). Different perpetrators have been identified in the literature such as coaches, peer athletes, and medical staff (Alexander et al., 2011; Vertommen et al., 2013). Higher prevalence of male perpetrators has been observed (Kirby and Greaves, 1997; Vertommen et al., 2013; Johansson and Lundqvist, 2017). Coaches as perpetrators have been more studied and the magnitude of CSA perpetrated by them varies between 0.2 and 9.7% (Parent and Fortier, 2017). Because of the importance of the coach role and their proximity with athletes, it seems important to better understand the experiences of the coach-athlete relationship. Moreover, some studies show that the impacts of CSA are more severe when the perpetrator is in position of authority (e.g., a coach; Leahy et al., 2008).

Experience of CSA from a coach in the sport context is particular as it implies dimensions of power, influence, trust, and dependence (Johansson and Lundqvist, 2017). This critical relationship makes athletes more vulnerable to being exposed to CSA (Gaedicke et al., 2021). Some coaches may abuse this close and trusting relationship to create physical proximity with the athlete (Brackenridge and Fasting, 2005; Owton and Sparkes, 2017); however, this can make it difficult to identify the boundaries of the relationship. This particular dynamic makes it harder for athletes to recognize when an abusive relationship is being established (Gaedicke et al., 2021). Coaches may use grooming, defined as “the process by which a perpetrator isolates and prepares an intended victim” (Brackenridge, 2001, p. 35), to get closer to the athlete and commit CSA without it being noticed and disclosed. The disclosure of CSA received some attention in the sport literature, but not with great details about the process survivors go through around this specific question. Understanding this “process” could greatly help better support survivors in their journey through healing.

### Disclosure of CSA in Sport

Although a substantial number of youths, both inside and outside of the sport context, have been victims of CSA, studies suggest that many do not disclose their experience during childhood (London et al., 2008; Collin-Vezina et al., 2015; McElvaney, 2015; Jeong and Cha, 2019). For many victims, it takes more than two decades from the beginning of the abuse to tell someone about it and nearly three decades to have in-depth discussions (Easton, 2012). Delayed or lack of disclosures are alarming since studies have shown that the healing process usually starts with disclosure (Chouliara et al., 2014; Jeong and Cha, 2019). At this point, the victim can seek social and professional support (Collin-Vezina et al., 2021). One explanation of non-disclosure can be attributed to the lack of understanding of children on what CSA constitutes (Schaeffer et al., 2011). Some athletes reported that they were not always conscious that they were experiencing CSA at the time of the abuse (Owton and Sparkes, 2017). This may be explained by some athletes thinking that the relationship they have with their coach is a love story (Papathomas and Lavallee, 2012). They might find it flattering that their coach chooses them which allows them to feel special (Owton and Sparkes, 2017). Athletes also sometimes normalized sexual relations with the coach (Stirling and Kerr, 2009; Parent et al., 2016), perceiving it as consensual (Toftegaard Nielsen, 2010).

The “normalization” of CSA by victims is not specific to the sport context. In a study from Lahtinen et al. (2018), 51% of participants who reported a sexual experience with someone at least 5 years older did not self-label their experience as CSA. Moreover, 33% of the participants were not sure how to describe their experience, while some even considered it flattering (Lahtinen et al., 2018). In line with these results, Stige et al. (2020) conducted semi-structured interviews with 11 adult survivors of CSA to explore how they came to understand that they had been victims. Their results suggested that the process to understanding is long, complex, and individual. It is influenced by the ambiguity of the memory (i.e., experiencing flashbacks), the language of the body (i.e., high level of distress), and the encounter of an observant (i.e., challenges feeling emotionally close to others; Stige et al., 2020). These studies suggest that some CSA victims only realize many years later that they have been sexually abused, which may represent one barrier to disclosing their experience.

Research on disclosure of CSA is scarce within the sport context. Victims mentioned that they kept silent on the sexual relationship with their coach due to fear of jeopardizing their career (Moola and Krahn, 2017; Roberts et al., 2019) or of not being believed (Stirling and Kerr, 2009; Parent, 2011). Victims also thought that the position of authority of the coach contributed to their silence (Moola and Krahn, 2017; Roberts et al., 2019). Hartill (2014) conducted narrative interviews with two victims of boyhood sexual violence in male sport. The participants mentioned that they disclosed their experience for the sake of other athletes that might be going through similar experiences and to open the door for them to speak out. Owton and Sparkes’ (2017) case study of CSA in sport yielded similar results. The victims believed that participating in research where they shared their experiences could help other victims to speak up. Many studies outside the sport context have further investigated the barriers and facilitators to the disclosure of CSA (Collin-Vezina et al., 2015; Lahtinen et al., 2018). Results show that fear of not being believed (Alaggia, 2005; McElvaney et al., 2014; Morrison et al., 2018), shame and self-blame (Alaggia, 2005; Hartill, 2014; McElvaney et al., 2014; Collin-Vezina et al., 2015), concerns for self and others (Collin-Vezina et al., 2015; Morrison et al., 2018), and feelings toward the abuser (Morrison et al., 2018; Halvorsen et al., 2020) can hinder disclosure. Family-related factors such as dysfunctional communication and hostile environments can also suppress disclosure (Collin-Vezina et al., 2015; Alaggia et al., 2017). Studies suggest that age is a strong predictor of disclosure (Alaggia et al., 2017), where older age increases the propensity of disclosing (Collin-Vezina et al., 2015). Settings that provide an opportunity to share, such as interviews, are contextual factors that can facilitate disclosure (McElvaney et al., 2014; Alaggia et al., 2017).

Despite the known consequences of CSA, studies outside the sport context have shown that victims can heal from the traumatic experience (Jeong and Cha, 2019). Healing is “characterized by activity resulting in improved functioning as balance is achieved. People are viewed as active agents in their healing process, and their healing is not something that is done to them—it is something that takes place within them” (Arias and Johnson, 2013, p. 823). Healing is influenced by multiple factors such as secure and supporting relationships (Arias and Johnson, 2013; Easton et al., 2015), spirituality (Knapik et al., 2008; Draucker et al., 2011; Arias and Johnson, 2013), therapy (Arias and Johnson, 2013; Easton et al., 2015), formalizing a complaint or taking legal actions against the perpetrator (Chouliara et al., 2014; Easton et al., 2015), accepting that CSA is part of their life (Arias and Johnson, 2013; Jeong and Cha, 2019), and shifting blame (Draucker et al., 2011; Arias and Johnson, 2013; Chouliara et al., 2014). This final factor has been recognized as an important component in the healing process of many victims. It is a positive cognitive process that allows the victim to place the blame back on the perpetrator (Arias and Johnson, 2013). At this point, victims recognize that they are not responsible for what happened. It helps them to make sense of the abuse and reduce their shame and guilt. Shifting blame may represent a turning point toward healing from the traumatic experience (Draucker et al., 2011; Easton et al., 2015). In the sport context, one victim explained how labeling her experience as CSA helps her switching blame and release her from responsibility (Papathomas and Lavallee, 2012). She also expressed how sharing her experience of abuse with others confirmed that she was not to blame. The reactions she received validated that she was not responsible for her experience. As we can see, disclosure and healing are two process that are clearly linked. However, we do not have much detailed empirical knowledge of those two processes and how they are linked when it comes to survivors of CSA in sport. Understanding those processes could greatly help better support survivors in their journey through healing.

### Aim of the Study

A victim’s understanding of the sexual abuse, factors that influence disclosure and healing processes have been well studied over the past years in the general population, but little is known within the specific context of sport. Few studies in the field of sport have employed a narrative analysis and use the own words of the victim athletes to describe their experiences (Fasting and Sand, 2015). Experts have called for the need of qualitative research to have a deeper understanding of sexual violence in the sport context (Vertommen et al., 2017). Until now, most of the qualitative research have documented the grooming process (Owton and Sparkes, 2017; Bisgaard and Støckel, 2019). We therefore understand how CSA in sport occur but do not know much of the disclosure and aftermath of it. To our knowledge, there are a lack of studies following an athlete’s journey over time of CSA. This represents a major limitation since research shows that understanding, disclosure, and healing are not discrete events, but rather ongoing and dynamic process (Chouliara et al., 2014; Alaggia et al., 2017; Stige et al., 2020). Hence, the aim of the study is to present a single case study of CSA in sport to better understand the global experience over time from the perspective of an athlete.

## Materials and Methods

A narrative inquiry approach was adopted to conduct a case study based on a retired athlete’s lived experience of sexual abuse in the sport context. A unique narrative case study allows an in-depth and detailed understanding of the lived experience of a particular single case (Fortin et al., 2015; Creswell et al., 2018). There is “a fundamental belief in narrative research that stories mean something, and that they are a rich resource for trying to understand people and the world around us” (Andrews, 2020, 2). Stories not only represent a way to make sense of one’s experience, but also to meaningfully share them with others (Smith, 2010; Andrews, 2020).

Our journey in the current study began when the participant, Eva,^1^ contacted one of the authors (SP) after hearing a radio interview about violence in sport. Listening to the interview represented a turning point for Eva, since it was the moment she decided to tell her story with the goal of contributing to research. Narrative inquiry entails the *storying* and *re-storying* of lived experience (Creswell et al., 2018). This represents a relational process, where both interviewer and interviewee co-construct the direction and meaning of the story (Creswell et al., 2018; Andrews, 2020). For example, when asked to choose, Eva preferred to be identified as a survivor and not as a victim of sexual abuse. This question was important given as both terms are used in the literature, but it also provided her with additional control over her narrative.

### Data Collection

Two non-structured interviews were conducted with the participant in September and October 2019: the first lasting 86 min and the second 92 min. The interviews were audio recorded and transcribed verbatim. A third interview was proposed to Eva during the winter of 2021. The initial analyses revealed an evolution in her discourse and the researchers believed that an additional interview may reveal other meaningful information. Eva accepted to participate in a third interview which lasted 114 min. This study was approved by the University’s Research Ethics Board (certificate # 2018-204/12-06-2019). In terms of ethics, Eva signed a consent form where she endorsed the publication of her story in a scientific publication. Also, many precautionary measures were taken to ensure her wellbeing. Indeed, the conduction of the interviews was done by a senior researcher (SP) who had studied sexual abuse in sport for many years. Also, Eva was followed by a psychologist before, during, and after all interviews. We also gave her a list of other resources she could contact if needed, including a psychologist who worked with the research team. In addition, the interviewer (SP) was in contact with her before and after each interview to be informed of Eva’s wellbeing. Moreover, great care was taken to ensure that no identifying information about the participant (or the people involved in this case study) was included in the article. An additional precaution was taken in regard of the research team (co-authors), namely, by the use of a confidentiality agreement for all co-authors who accessed the data. The co-authors never met Eva in person and were not aware of her real name.

Qualitative interviews provide rich and detailed stories and provide insight into the participant’s emotions and meaning of their experiences (Smith and Sparkes, 2016). Unstructured interviews allow survivors to present their story in their own words and decipher it while disclosing it. The athlete was free to say anything she felt ready to disclose in the sequence she wanted (Fortin et al., 2015) and had a sense of control over the interview (Smith and Sparkes, 2016). This type of interview was chosen to allow Eva to speak freely about her experience. This choice was also based on other case studies conducted in sport that used a narrative approach (Hartill, 2014; Bisgaard and Støckel, 2019). Specifically, after Eva manifested herself to one of the authors (SP) expressing an interest to contribute to research by sharing her experience, SP and Eva met together to explore her intentions and desire to share and what this could implicate from a research and ethical perspective. After this meeting, Eva decided to officially engage in the research process and signed the consent form. Therefore, the conduction of the interviews happened after some contacts with Eva, meaning that SP and Eva knew each other before the interviews. Given that the goal of Eva’s process was to share her story, this allowed to dive more directly in the subject of the interview. Also, SP asked Eva to prepare for the first interview by reflecting on a chronological timeline of her story, which became the starting point of the first interview. The interviewer’s role was then to ask for precisions and clarifications in a supportive and empathetic way. The first and the second interviews were ended by Eva, when she felt it was time to stop (no more time available or because of the fatigue that it created for her). The starting point of other interviews (2 and 3) was always about additional information that she wanted to share and that we did not have time to address. Considering that some time passed between each interview, Eva had time to reflect and was free to speak about what she wanted to share. This methodology enabled the interviewer to respect what was important for Eva to share. It also brought a very useful way of understanding her experience without having pre-conceptualized or oriented questions from a research standpoint.

### Data Analysis

Inductive thematic analysis was conducted (Braun and Clarke, 2013). Six steps were followed as: (1) familiarization with the data was achieved by listening and reading several times all verbatim, (2) coding was conducted by two coders concurrently, (3) the two coders then searched for patterns in the codes to create themes, (4) themes were reviewed by all four authors, (5) themes were then named and defined, and (6) then written up to tell the story. Data codification was organized using NVivo 12. The reliability of the results was confirmed by the co-coding of the data by two researchers in the team (Guba, 1981; Loh, 2013). They proceeded to analyze the data together. At some point in the analysis, the coders referred to the literature to frame the findings and found an article suggesting a theoretical model on healing from CSA (see Arias and Johnson, 2013). This model helped them realize that the participant has been and was still going through different processes in her healing journey. From the first to the third interview, Eva’s narrative is marked by an evolution as she has been attempting to decipher her experience during the research project. The four authors felt it was important to highlight this evolution in the results. The coders came up with the term “pathway” to illustrate the principal and distinct phases of the survivor journey. Themes and code were then discussed between all the team researchers until unanimity was reached. The involvement of all authors in the data analysis process ensured the rigor and trustworthiness of the results. To ensure the accuracy of interpretations, themes and codes were validated in a fourth meeting with the participant at the end of the analysis (Guba, 1981; Loh, 2013). Minor adjustments were made based on the participant’s feedback.

### Meeting Eva

Eva is a Canadian retired female athlete aged 36 years at the time of the study. She had practiced her team sport at the provincial level for 18 years. Eva had been sexually abused by her male coach, Allan, for 6 years, starting at 14 years of age. The cycle of abuse began when Allan started to give her rides to training. The abuse then started in her coach’s car and continued in the basement of the coach’s parents and in competition. The abuse included primarily oral sex and mutual masturbation with digital penetration. At the time of the abuse, Eva was in love with Allan. She did not realize that the relationship they had was abusive. As requested by Allan, Eva kept quiet about the sexual relationship with her coach for 20 years. She is now married and has two kids.

## Results

Eva’s story illustrated how she managed the abuse experienced as a child. In her own words, this was long and complicated as:

It is like if you take your life, your puzzle of a thousand pieces and you just shake the box, then you open it again, you do not know how many pieces there are, you do not know where your corners are, and so it is really difficult but at the same time, really liberating.

Her non-linear pattern of discourse showed Eva actively engaged in reflection and, to paraphrase her, in putting the pieces back together. Through re-storying (Creswell et al., 2018), we identified three pathways: (a) pathway to understanding, (b) pathway to disclosure, and (c) pathway to healing (see Table 1). These pathways represent distinct processes where understanding involves making sense of one’s experience of abuse; disclosing involves acting upon one’s experience; and healing involves overcoming one’s experience. However, the three pathways are intertwined. It would be difficult to heal before understanding one’s experience, for example. While actions of disclosure can help with sense-making, it can also facilitate the healing process by engaging with resources. Finally, the pathways are dynamic and iterative in the sense that the survivor explained how she had been, and is still, going back and forth between pathways. Understanding, disclosing, and healing should be observed as distinct yet complementary processes by which Eva was putting back the pieces of the puzzle her abuse represented back together.

**Table 1:** Athlete’s narrative on sexual abuse in sport.

Main themes and sub-themes	Definitions
Pathway to understanding	Process of making sense of the abuse
Ambiguity	Love-abuse duality in the survivor interpretation of her experience
Language of the body	Symptoms that communicate to the survivor that something is wrong
Triggering elements	Elements that helped the survivor to better understand her experience
Labeling the abuse	Act of putting words on one’s experience
Pathway to disclosure	Process through which the survivor began acting upon her experience of abuse and sharing her evolving understanding with others
Barriers to disclosure	Hindering elements to disclosure
Pathway to healing	« Activity resulting in improved functioning » (Glaister, 2001)
Acceptance	Cognitive processes of de-guilt and de-responsibilization
Steps of active healing	Steps the athlete takes to face her experience and become an active agent in her healing process
Strengthened resilience	Increased control and power over one’s life and strengthened sense of oneself

### Pathway to Understanding

This pathway illustrates the process of how Eva came to make sense of the abuse she experienced as a child. It shows the evolution in the awareness and interpretation of her own experience but also of sexual abuse in general. Eva came to the first interview being unsure if what she had experienced could be considered abuse and came out of the third interview considering herself to be a survivor. She described feelings of ambiguity that made it difficult to reconcile what she experienced with her conception of sexual abuse. Eva explained how certain events in the media and in her life experiences became triggers that moved her toward understanding. Furthermore, the fact that she was educated made her more comfortable with researching information, helping her to better understand her experience. Her body language also increased her awareness of the trauma she had lived. Through making sense of the ambiguity and her body language, and with the triggering aspect of certain events, Eva eventually labeled her experience as abuse. She also described how she came to a place of acceptance and lessened her sense of guilt.

#### Ambiguity

A strong sense of ambiguity was present in Eva’s discourse, especially in the first interview. She had mixed feelings about her abuser and concerning her experience of abuse more broadly. Eva confided that she had been in love with Allan and that she had hoped to become his girlfriend. At the same time, she realized that their relationship was not normal because he insisted on keeping it secret, and as she stated when “you have to keep something secret, it’s usually because it’s not right.” She explained that part of her thought that this was because of her being underage and that he would legitimize their relation when she turned 18, or as she metaphorically said, that he would “take her out of the basement.”

Eva’s conflicted feelings about her abuser influenced how she interpreted the abuse. Since she loved him and wanted to please him, she never offered any resistance. This was contrary to what she conceptualized as an abuse, and it led her to doubt her own experience. For her, sexual abuse was something violent, direct, and implied penetration:

In the sense that sexual assault necessarily means rape. That there was physical coercion, that there was—that the person said, “No” you know, a sexual assault is not noiseless. Whereas what I experienced was really silent.

Her doubts were so strong, that at the end of the first interview, Eva asked the researcher, crying: “Well, basically I need you to tell me whether or not… if this really was abuse, because I am mixed up.” More than a decade after the abuse, in an interview where she sought out to share her story of abuse, she needed to validate her understanding.

#### The Language of the Body

During her life, Eva’s body expressed the suffering related to the abuse from which she tried to distance herself mentally. She mentioned many symptoms, such as constant fatigue and frequent headaches. In the period directly following the end of her relationship with Allan, Eva began drinking heavily. She used alcohol to numb her feelings and for some time, she was not capable of being intimate sexually if she was not intoxicated. There came a point where she met a man with whom she chose to settle down and have children, which was when she decided to quit drinking. She also began therapy to address mental health issues, which she described as “pervasive unhappiness.” However, she never discussed her sexual abuse with her psychologist at that time since she did not think that it was the cause of her difficulties. Her body was sending her signs that she was not yet able to interpret in relation to her experience of abuse. She explained as: “I never went straight into a psychologist’s office and said, ‘I was a victim.’ You know, it came out differently.” After each of the first two interviews, Eva had significant physical pain which she said was “as if I had been in a car accident.” The intense pain appeared after recounting her experience of abuse in detail for the first time in her life which ultimately helped her realize that she really had been abused.

#### Triggering Elements

Media coverage of high-profile sexual abuse cases has increased over the last decades. Eva recalled that the first case that triggered her happened in 2008 when a public figure denounced her personal experience of CSA with a well-known man in the music industry. The fact that the story was very similar and came out publicly as sexual abuse legitimized Eva’s own ambiguous and confused feelings about her relationship with Allan. This specific case made her realize that what she had experienced was abuse. The media then helped her better understand her personal experience by identifying with other survivors.

Eva is an educated woman who explained that when she needed to better understand her experience, she looked for information at the library or on specialized websites. She described her quest for information as a coping mechanism. For example, in her process of understanding, she wondered why she had never confided in her doctor, she explained as: “I took out all the books on sexual assault available at the library and I found that the most recent statistic is 2 to 9% of people who talk about it to their doctor.” Eva also had triggers through her professional career where she learned about aspects related to sexual abuse, such as the importance of secrecy and of rewards given by abusers. It made her realize that her abuser “knew he wasn’t right.” Getting information was a way for her to better understand and normalize what she had experienced.

#### Labeling the Abuse

Her pathway to understanding eventually led Eva to label her experience as abuse. The increasing awareness about sexual abuse and about the importance of consent in the last decades helped her to understand what happened and describe her experience in the right words. In retrospect, she felt there was not enough education to provide words to describe what she was experiencing.

It just seems so obvious to me now. (…) But twenty years ago, well… I’m trying to put myself back, you know, twenty years ago it wasn’t like that at all, at all, at all. It’s very hard to put it in context. Twenty years ago, nobody was talking about consent.

Her conceptualization of sexual abuse was embedded in the societal context. Eva mentioned that 20 years ago no one was suspicious of sexual abuse between a coach and an athlete, saying “nobody thought it existed.” Although the evolution of knowledge about sexual abuse in society helped her own understanding, Eva says she still feels conflicted at times. Throughout the interviews, she referred to her “child mind” competing with her “adult mind.”

Well, you know, that story has been written in my head as a child. I do not know how—it is there, it is compartmentalized, you know, and now I can put adult words to it. And language has evolved a lot in the last 20 years.

This quote illustrates that Eva’s conception of CSAs has evolved over time. The understanding of her experience when she was younger differs from her current understating. The recognition if CSA in sport in society and the evolution of vocabulary has helped her in her process. Getting older and gaining life experience has also shaped her “adult mind.”

### Pathway to Disclosure

This pathway documents the process through which Eva began acting upon her experience of abuse and sharing her evolving understanding with others. She had discussed her abuse with very few people, namely, her husband and three friends, but never with her psychologist whom she continued to see periodically over the years. She had always stayed vague in her statements by using euphemisms such as “bad experiences” or “inappropriate behaviors.” As mentioned previously, Eva disclosed her experience in detail for the first time through the interviews.

#### Barriers to Disclosure

Inherent to this slow and cautious process, Eva identified several barriers that limited or delayed her disclosure. Some of these barriers were relational, such as the climate in her family that did not promote open communication, or the reactions of loved ones. One barrier was specific to her fear of losing access to her sport if she reported the abuse. Finally, two other barriers were related to the apprehension of being labeled as a victim and her related distrust of the criminal justice system.

Eva explained that the climate in her family never promoted open communication about sensitive issues. On the contrary, it was “an atmosphere of simmering conflict (…) we never shouted, but we never spoke either” which did not make her feel secure to discuss her discomfort and mixed feelings toward her coach. Furthermore, her parents knew and seemed to appreciate Allan who sometimes chatted with them on the phone when calling Eva. This made her feel like she did not have a “space to express myself.”

During the period the abuse was taking place, Eva feared she could lose access to her sport if she reported her coach. She deeply appreciated her sport and competed at high levels which left little alternative if she had wanted to transfer to another team. Ultimately, Eva felt that if she had spoken up about the abuse, this would have led to her having to stop practicing, to her teammates turning against her and to losing her place on the team. She described this situation as a “double abuse, because if you speak, you lose so much.” She also cared about her coach’s reputation, keeping her quiet by fear of tarnishing it.

When she eventually began to confide in some of her loved ones, Eva said she did not receive the reaction she needed from them. She explained that when the subject of sexual abuse was mentioned, “people fall into anger very quickly.” Although these reactions were directed toward the abuser and not toward Eva, she did not feel that there was authentic listening. While the context of public denunciations led to increased knowledge about sexual abuse, Eva felt it also led to a lack of understanding of why people do not speak up. Since the few times where she began discussing her experience led to people becoming angry, she closed up and kept vague about the nature of the abuse.

Eva perceived that disclosing her experience of sexual abuse would have categorized her as a victim. She mentioned that, in a sense, disclosing her abuse would make it real: “As long as we do not say it out loud, and we do not discuss our experience, to admit that we have been victimized by a person [sobbing] well if you do not say it, you are not [a victim].” Eva mentioned that once a person is labeled a victim, it stays with her for the rest of her life. This fear of being labeled as a victim is related to her distrust of the criminal justice system where she observed other victims of sexual abuse being “savagely discredited” and stigmatized. The notion of innocent until proven guilty places the burden of proof on the victim who “has to prove that she has really been a victim.” For Eva, that would have felt as being re-victimized. She felt this very demanding experience was not worth it since the sentences were not severe enough and that the accused often got away with a “slap on the wrist.”

During the period that the interviews took place, however, Eva’s discourse showed an important evolution in her perception of these barriers. Evidence of her moving through her pathway of disclosure, Eva explained during the third interview that she had shared her story of abuse with her husband including all the details she had previously left out. She also communicated with her psychologist to specifically address her experience of abuse and was engaging in civil judiciary actions against Allan. This disclosure to her husband placed stress on their relationship and brought back some difficulties with their intimacy, but Eva felt this was an important step toward healing.

### Pathway to Healing

Through her understanding and disclosing, Eva began moving toward healing. She described being largely motivated by her children: “In my head and in my heart, I often say to myself that I had my daughter to *survive* and then my son to *live*. It’s a strong impulse.” The pathway to healing was described as a positive, yet bumpy road that included difficulties and challenges. Eva admitted being tired by the actions she had been taking: “It’s very tiring to do this, but I think it’s a fatigue related to releasing tension. I do not think the fatigue is setting in. I think the fatigue is coming out.” Eva took many active steps to allow her to heal, such as participating in the current research process, seeking therapy to specifically address the abuse, and confronting her abuser. She also discussed how the process of healing, namely, through her taking back power, had developed her sense of resilience.

#### Acceptance

This distinction between her child mind and her adult mind represents a metaphor she used to illustrates her understanding that what her abuser did was wrong, although she was not able to see it that way when she was a child. While Eva has journeyed on her pathway to understanding to a place where she now labels her experience as abuse, her process of acceptance is ongoing. She described it as a need to reprogram herself.

I am reprogramming myself; I say to myself “he was more than 5 years older, he had the authority and he touched you over your clothes, he had no right to do that” I repeat that to myself. Because I have done the opposite so much, it is like I have to reprogram myself.

Eva explained that the more she accepted her experience, the more she developed self-compassion that replaced her initial feelings of shame and guilt. To continue on this path, she recognized that she also had to come to terms with the romantic feelings she had for Allan. Eva explained that in a sense, she went through a breakup yet has never felt the heartbreak or romantic grief that could help her move on from the relationship. In a recent session with her psychologist, she realized that she was not only sexually abused, but also emotionally abused. She referred to this as a “love abuse.” This led to painful physical symptoms and eventually to a sense of having surmounted her romantic feelings.

Days later [the session with the psychologist] I was hurting, I was hurting, and I was just crying all the time. (…) Like an old heartbreak that resurfaced. Once I understood that it was over. Well, I was tired, but after that I did not have romantic feelings and I am not finding excuses for him anymore. It is like it is been evacuated of the equation.

#### Steps of Active Healing

The current research played an important role in Eva’s healing process as she stated that it was where her healing started. She explained that it had become important for her to do something with her experience, yet she did not want to engage in legal procedures. She chose to contact the researcher because she felt she could have control over the situation, which she said would not have been the case in the criminal justice system.

I realized that it was a safe, confidential environment where you would be able to receive what I had to say (…) the idea of participating in research, well, it did not imply anything. In the sense that it could end there. If you go to the police station to report, things are out of your hands. You do not have control over your situation whereas here, well, I decide a lot of what happens and for me it was necessary to move forward with this.

Participating in research has been a way for her to validate and normalize her experience, while gaining a clearer understanding of her situation. Eva also appreciated that recounting her story would “serve a purpose” and that it was a way for her to contribute to research in this area.

Although she had been actively engaged in therapy in the past, Eva had never addressed her experience of sexual abuse. After the first two interviews, Eva made an appointment with her psychologist whom she had not seen in some time. This allowed her to gain a better understanding of her experience and related feelings, to learn coping strategies, and to make sense of her own life path. Eva compared the healing supported by her psychotherapy to physiotherapy for a person who had suffered a physical injury.

Another action Eva took on her healing journey was to confront her abuser. She explained that this allowed her to regain a sense of control by placing the responsibility back into him. Eva described an event where she invited Allan to a restaurant and told him “I never want you to write to me again, I never want you to call me, I never want you to call my parents. I never want to have contact with you again. And you were not always right.” Recently Eva had engaged in a civil procedure to confront Allan more formally, which she would not have considered before the research. She explained that she decided that she wanted “to say [to him] what I had not been able to say in the end (…) There is this, this, and this, it belongs to me. You took it from me.”

Eva found the legal procedures exhausting and time-consuming, but she thought it would lead her to “have more freedom to be. To have less time to devote to my healing, to be more available in my love life, to be more, to have a more open heart with my children.” However, she remained concerned as: “there is a part of the aftermath that worries me, what will my condition be afterwards? I think it will be better, but how much better will it be?” This demonstrate how the healing process is a bumpy road for Eva.

#### Strengthened Resilience

Eva’s pathway to healing led her to increase her control and power over her life. She described having a strengthened sense of herself that allowed her to stand up for herself in uncomfortable situations. She gave an example of where she confronted her boss after he made comments of a sexual nature which made her feel “super powerful.” Eva said that she now recognized her self-worth and was better able to set her limits. She described that she was much more comfortable dealing with authority figures in her professional or personal life. Eva said that she also felt empowered to speak up for other athletes and women who could not, for different reasons, do it for themselves. Ultimately, Eva described her ongoing journey of healing and shared her hopes for her future.

Well, when I came here, I reached out like “I think I was maybe a victim” and after these two interviews, I came out a survivor, but now I just want to be alive. I want it to be fixed and to move on with my life, not to survive. For me, that is my goal. And one’s life is filled with desires, projects, it is about projecting myself in time, not just going through the days.

## Discussion

The purpose of the study was to better understand the experience of CSA in the sport context from the perspective of a survivor-athlete. To do so, a narrative single case study was conducted with a female retired athlete who was a victim of CSA by her coach for 6 years. It took the athlete 20 years to disclose her complete experience of abuse. This timeline aligns with research demonstrating that many children do not disclose their experience before adulthood (Hébert et al., 2011; Easton, 2012; Collin-Vezina et al., 2015). Through thematic analysis, the authors identified three major themes in the survivor’s narrative: (a) the pathway to understanding, (b) the pathway to disclosure, and (c) the pathway to healing. These themes are discussed in detail in the following sections within the context of the broader literature.

Eva’s pathway to understanding represents the events that led her realize that she had been sexually abused by her coach when she was a child. The fact that it took her many years to understand what happened and describe her experience in the right words is consistent with other victims’ narratives (Lahtinen et al., 2018). Eva’s pathway to understanding began when an artist publicly denounced her story of sexual abuse. Eva mentioned that she became aware of the many similarities between this public story and the relationship she had with her coach. This triggering element is consistent with other survivors’ stories. In sport, one athlete stated that she came to understand that she had been sexually abused by her coach when she was exposed to a similar situation on a television show (Papathomas and Lavallee, 2012). The exposure to the parallel of her own experience helped her realize that she was manipulated and that she was not to blame. The presence of manipulation from the coach is also present in many other survivors’ narratives and is linked to the grooming process (Brackenridge, 2001). The coach progressively goes beyond the limits of the interpersonal relationship with the athlete. This allows him to determine if it is safe for him to move on to the stage of sexual abuse without the athlete resisting and reporting it (Bisgaard and Støckel, 2019). Manipulation can take the form of the coach giving compliments to the athlete, giving gifts, offering special treatments, or inviting the athlete to their home (Fasting and Sand, 2015; Owton and Sparkes, 2017). Those privileges are a way of gaining the athlete’s trust and can explain why athletes often perceive the situation as a love story and not abuse (Papathomas and Lavallee, 2012; Fasting and Sand, 2015). The ambiguity in Eva’s understanding of her experience could be explained by the grooming from her coach. As mentioned previously, it could also be explained by the normalization of the coach-athlete romantic relationship in the sport context (Stirling and Kerr, 2009; Parent et al., 2016).

Barriers to disclosure exposed in our study are consistent with those found in the literature on CSA (Alaggia et al., 2017; Moola and Krahn, 2017; Roberts et al., 2019; Halvorsen et al., 2020). The fear of losing one’s sport is a specific barrier in the sport context. As in Eva’s case, many survivor-athletes are afraid to denouncing their abuse because they fear it would have a negative impact on their career (Moola and Krahn, 2017; Roberts et al., 2019). Other survivor-athletes’ narratives highlight that sexual violence in the sport context is something they learn to accept and deal with Fasting et al. (2007). They state that the cost of fighting is not worth the chance of jeopardizing their performance success. Sexual abuse is embedded in a sport culture where athletes are expected to show resilience in the face of adversity (Fasting et al., 2007).

Eva even compared CSA in sport context to CSA within the family. She said that because most of the time your sport team becomes like your family, CSA in sport context is kind of incestuous. The fear of losing one’s sport could then be compared to the fear of the impact disclosure could have on the family dynamic when CSA occurs in-between family members. For example, Eva exposed her concerns about the impact disclosure could had have on her team. She said that she would not have wanted it to tarnish her coach reputation, that her teammates turn against her, that it would have tear her team apart, or that it would have harm her sport. Those are the types of concerns victims of CSA within family also express. Children are scared that disclosure would result in the abuser getting into trouble, that the family would suffer from it, or that it would break up their family (McElvaney et al., 2014). The incestuous nature of sexual violence from a coach has also been highlighted by Brackenridge (1997).

In the two first interviews, Eva stated that she had never been able to fully disclose her CSA experience to anyone. She spoke briefly about it to her husband, two colleagues, and a friend, using euphemisms and remaining vague. Eva’s narrative illustrates how loved ones’ reactions can impact the disclosure process. Hateful reactions toward the perpetrator can cause victims to turn inward and stay vague in their disclosure. The use of euphemisms when talking about one’s experience of sexual abuse in sport is present in other survivor-athletes’ narratives as well (Papathomas and Lavallee, 2012). Not being able to properly name the act can be explained by the ambiguity survivors still experience even years after its occurrence. Consistent with Eva’s feelings, it is still difficult for many survivor-athletes to not blame themselves for what happened (Fasting et al., 2007; Papathomas and Lavallee, 2012). This can make it difficult to use the word “abuse” when discussing their experience.

Concerning the third pathway, our results align with the theoretical model of healing from CSA (Arias and Johnson, 2013). This model illustrates the significance of supportive relationships and positive internal characteristics in the healing process. This model also suggests that a decision point can act as a trigger to active healing, such as having kids. This is consistent with Eva saying, “In my head and in my heart, I often say to myself that I had my daughter to *survive* and my son to *live*. Those are big impulses.” According to Arias and Johnson (2013), supportive relationships, positive internal characteristics, and turning points (e.g., having children) lead to active healing. This model considers active healing as multifaceted. In line with the present study, therapy, life experiences, attributing blame to the abuser, confronting the abuser, empathy, and compassion were sources of active healing for Eva. Also consistent with our study, acceptance and strengthened resilience are results of active healing. This suggests that the theoretical model could be applied to CSA in sport.

Eva’s pathway to healing can also be linked to the concept of post-traumatic growth. Post-traumatic growth is defined as “the individual’s experience of significant positive change arising from the struggle of a major life crisis” (Calhoun et al., 2000, p. 521). By her actions, Eva is taking control of her healing process. Confronting the abuser to return the blame, acceptance, adaptive coping strategies (i.e., education and normalization), and taking back power are concepts that have been linked with post-traumatic growth (Ullman, 2014; Hartley et al., 2016). Post-traumatic growth leads to positive change (Woodward and Joseph, 2003; Ullman, 2014) which, in the case of Eva, can be illustrated by her strengthened resilience, a better self-perception and new perspectives in life.

The non-linearity and dynamic aspect of her pathways are consistent with other CSA victims’ pathways. Qualitative studies conducted with CSA victims have shown that disclosure is a lifelong dynamic process that does not take place in a single event (Alaggia et al., 2017). Studies have also shown that healing is a non-linear, ongoing, and dynamic process. The pathway is marked by a “back and forth” and is multifaceted (Draucker et al., 2009; Chouliara et al., 2014). Eva is not the only CSA survivor for whom the healing process is not always easy. Previous studies on victims of sexual abuse have also showed that healing is a challenging and tedious journey (Draucker et al., 2011).

### Practical Implications

#### Education and Clearer Boundaries in the Coach-Athlete Relationship

The pathway to understanding demonstrates that CSA victims do not always realize they are actually victims at the time of the abuse. The ambiguity between love and abuse has also been discussed in other studies (Papathomas and Lavallee, 2012) and shows the need for better awareness and education. Athletes need to know what the boundaries of a coaching relationship are. As observed in the present study and stated by multiple authors, boundaries of the coach-athlete relationship are not clear in sport (Stirling and Kerr, 2009; Bisgaard and Støckel, 2019; Gaedicke et al., 2021). This makes athletes more vulnerable to experiencing sexual abuse. More information should then be given to them and all actors in sport (parents, coaches, support staff, etc.) about sexual abuse in sport and boundaries in the coach-athlete relationship. This could not only help athletes and actors in sport recognize the abuse, but also help people around them intervene to stop behaviors of coaches that go beyond those boundaries.

#### The Role of Sport Psychologist and Other Sport Actors

Considering that romantic and sexual relations between coaches and athletes appear to be sometimes normalized (Parent et al., 2016), it is likely that athletes do not recognize the importance of discussing it with others. It then seems essential to address the subject directly with them. Since sport psychologists’ mission involves promoting the wellbeing of athletes and their protection (Kerr and Stirling, 2019), they seem to be well positioned to open the dialogue with athletes about possible problematic relationship with their coach. Even though sport psychologists occupy a privileged place in the promotion of the wellbeing of athletes, it is essential that every stakeholder takes responsibility to ensure an athlete’s safety. Sport organizations should then offer training to all stakeholders (i.e., athletes, coaches, and parents) about sexual abuse and sexual violence more generally. Sport stakeholders need to be better equipped to recognize the signs of sexual violence in sport. Educating them on definitions of sexual violence, risk factors, consequences, and boundaries in the relationship between coaches and athletes is of primary importance. It would help them identify problematic situations more quickly and easily (Kerr and Stirling, 2019).

In line with the post-traumatic growth theory (Ullman, 2014), sport psychologists could also help athletes to regain a sense of control over their experience and healing process. As our results indicate, shifting blame from the victim to the abuser is an important step toward healing. Therefore, sport psychologists should assist athletes in reinforcing that, as victims, they are not to blame which will help them grow from their experience (Hartley et al., 2016). They should also teach adaptive coping strategies to the athlete and support them in their steps to active healing (Ullman, 2014).

#### The Role of Research

Eva chose the context of research for her first disclosure. She also decided to first turn to research instead of the justice system. Qualitative research could be a potential avenue to help victims heal from their experience. It gives them the chance to talk about and make sense of their abuse in a safe space. By participating in research, victims feel like their experience could serve a purpose for helping others (Hartill, 2014; Owton and Sparkes, 2017). This sheds light on the positive and active role research can play for victims. This finding is in line with models of healing from CSA who identify altruism (e.g., helping other victims by speaking out and getting involved in victim’s organization) as a way of giving meaning to the abuse (Grossman et al., 2006) and a stage in the healing process (Draucker et al., 2011; Arias and Johnson, 2013).

#### Placing Victims at the Center of the Justice System

Eva considers that the criminal justice system is not suitable for victims of sexual abuse. Based on the public judicial processes of other victims, she came to consider that attending court is a form of re-victimization. This demonstrates the importance of reviewing the current justice system for victims. The recent announcement of Bill 92 in the Quebec National Assembly concerning the creation of a specialized court for sexual and domestic violence is consistent with this idea. This court will require judges to take a refresher program on the realities of sexual and domestic violence established by the Council of the Judiciary (Degré, 2021). This training will enable judges to better understand the reality of trauma experienced by victims. This bill is promising for sexual abuse victims as trauma-informed approaches “minimize the potential for harm and re-traumatization, enhance safety, control and resilience for all clients involved with systems or programs” (Public Health Agency of Canada, 2018). This court seeks to place the victim at the center of the process by bringing together all the psychosocial and legal services required by victims in one place (Degré, 2021). The trauma-informed approach appears necessary to offer victims the support needed not only for disclosure, but also for healing (Randall and Haskell, 2013). It also appears important that victims have access to alternative justice pathways such as the civil procedures that Eva used. In that sense, restorative justice has been suggested as a potential positive alternative, complementary, or supportive way for victims of sexual abuse (Naylor, 2010; Gavrielides, 2012; Koss, 2014). Compared to other forms of justice, restorative justice views crimes as a violation of people and relationships rather than a violation of the state (Government of Canada, 2021). The focus is places on repairing the harm. Restorative justice is based on communication between both parties (the victim and the offender; Government of Canada, 2021). Restorative justice appears to be a promising avenue to help victims to switch blame to the abuser (McGlynn et al., 2012).

## Limitations and Future Research

The current research is not without limitations. This is a single case study. Even though it provides a deeper understanding of the case, it does not guarantee that the findings apply to every athlete. Results should not be generalized for athletes of different countries, where cultures vary. Social and cultural norms regarding gender roles and behaviors, sexual violence, and romantic and sexual relationships may differ from one country to another, as do notions of consent (Kalra and Bhugra, 2013). Pathways could also be different based on the gender of the athlete. For men, being sexually abused by a male coach may question their sexual orientation (Hartill, 2014). This brings a particular element in their pathways which may then differ from women. Finally, the present study took place 20 years after the abuse happened which may result in memory recall bias on the events related to the abuse. However, this also gave access to a portion of the participant’s healing process in real time, which is novel in the sport-related CSA research domain.

Future research should explore the pathways with male athletes and athletes from different countries. To our knowledge, most studies on CSA in sport have been conducted with female athletes. It is thus important to understand whether male athletes are impacted similarly. It would also be important in future research to go beyond the grooming process in survivor-athletes’ narratives of sexual abuse and to explore their pathways more globally. Following a survivor-athlete’s journey over time would help to have a better comprehension of the understanding, disclosing, and healing process which in turn could help guide prevention and intervention programs.

## Data Availability Statement

The raw data supporting the conclusions of this article will be made available by the authors, without undue reservation.

## Ethics Statement

The studies involving human participants were reviewed and approved by University Laval Research Ethics Board (certificate # 2018-204/12-06-2019). The patients/participants provided their written informed consent to participate in this study.

## Author Contributions

AG contributed to the conception and study design and project management and drafted the manuscript. ES-P contributed to the data analysis and reviewed the manuscript. SR and SP contributed to the project management and reviewed the manuscript. All authors contributed to the article and approved the submitted version.

## Conflict of Interest

The authors declare that the research was conducted in the absence of any commercial or financial relationships that could be construed as a potential conflict of interest.

## Publisher’s Note

All claims expressed in this article are solely those of the authors and do not necessarily represent those of their affiliated organizations, or those of the publisher, the editors and the reviewers. Any product that may be evaluated in this article, or claim that may be made by its manufacturer, is not guaranteed or endorsed by the publisher.

## Footnotes

^1^For confidentiality purposes, names of individuals involved in the story were changed. The participant chose her pseudonym as Eva and that of her abuser as Allan.
